# mTORC1 signaling in hepatic lipid metabolism

**DOI:** 10.1007/s13238-017-0409-3

**Published:** 2017-04-22

**Authors:** Jinbo Han, Yiguo Wang

**Affiliations:** MOE Key Laboratory of Bioinformatics, Tsinghua-Peking Center for Life Sciences, School of Life Sciences, Tsinghua University, Beijing, 100084 China

**Keywords:** mTOR, mTORC1, lipogenesis, lipophagy

## Abstract

The mechanistic target of rapamycin (mTOR) signaling pathway regulates many metabolic and physiological processes in different organs or tissues. Dysregulation of mTOR signaling has been implicated in many human diseases including obesity, diabetes, cancer, fatty liver diseases, and neuronal disorders. Here we review recent progress in understanding how mTORC1 (mTOR complex 1) signaling regulates lipid metabolism in the liver.

## INTRODUCTION

Most organisms have evolved mechanisms to respond to dynamic environmental cues including nutrients, growth factors, and cellular energy levels for survival and growth. Mechanistic target of rapamycin (mTOR) integrates these environmental cues to modulate metabolic pathways for cell growth. Dysregulation of mTOR signaling has been implicated in many human diseases, including obesity, diabetes, cancer, and neuronal disorders (Cornu et al., 2013; Saxton and Sabatini, 2017).

**Figure 1:**
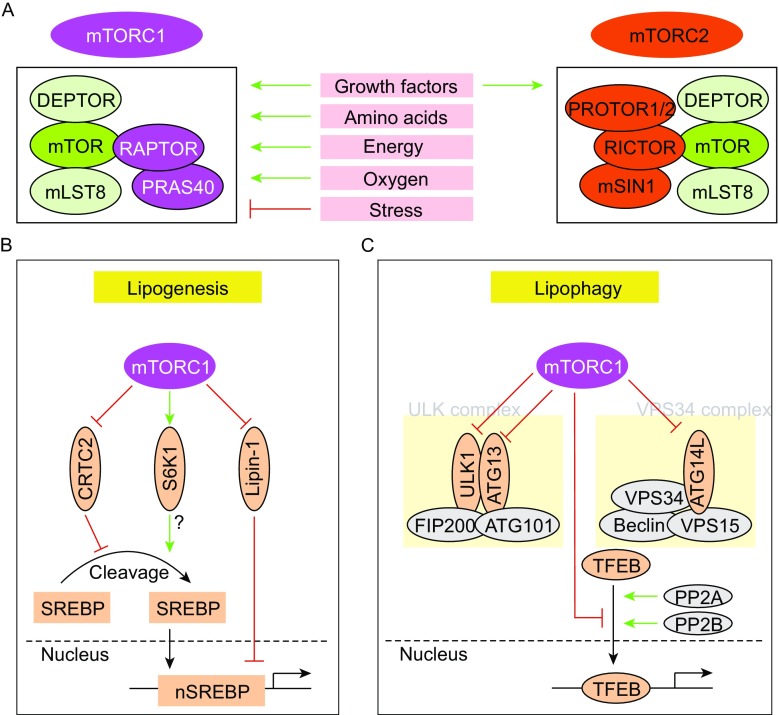
**mTOR signaling in hepatic lipid metabolism**. (A) The protein composition and key features of mTORC1 and mTORC2. mTORC1 responds to growth factors, amino acids, stress, oxygen and energy, while mTORC2 only responds to growth factors. (B) mTORC1 promotes SREBP-dependent lipogenesis through the phosphorylation of CRTC2, S6K1, and Lipin-1. (C) mTORC1 inhibits lipophagy by blocking autophagy initiation and attenuating lysosome biogenesis

TOR was identified in yeast genetic screens as the factor that confers resistance to the anti-fungal and immune depressant drug rapamycin, and its mammalian counterparts were identified shortly afterwards (Heitman et al., 1991; Cafferkey et al., 1993; Kunz et al., 1993; Brown et al., 1994; Sabatini et al., 1994; Sabers et al., 1995). mTOR is an evolutionarily conserved serine/threonine kinase that belongs to the phosphoinositide 3-kinase (PI3K)-related kinase family and exists in two distinct signaling complexes, mTOR complex 1 (mTORC1) and mTORC2 (Cornu et al., 2013; Saxton and Sabatini, 2017). Both mTORC1 and mTORC2 share four protein components, including the TOR kinase, DEP domain-containing mTOR-interacting protein (DEPTOR) and mammalian lethal with Sec13 protein 8 (mLST8) (Cornu et al., 2013; Saxton and Sabatini, 2017) (Fig. 1A). In contrast, regulatory-associated protein of mTOR (RAPTOR) and proline-rich AKT substrate 40 kDa (PRAS40) are specific to mTORC1, while rapamycin-insensitive companion of mTOR (RICTOR), mammalian stress-activated protein kinase-interacting protein (mSIN1), and protein observed with RICTOR-1 and -2 (PROTOR1/2) are only associated with mTORC2 (Cornu et al., 2013; Saxton and Sabatini, 2017).

mTORC1 is sensitive to rapamycin and promotes protein synthesis and lipid synthesis, as well as inhibiting autophagy and lysosome biogenesis in response to growth factors, amino acids, stress, oxygen levels, and energy status. These responses occur in part through the phosphorylation of mTORC1 substrates, including ribosomal S6 kinase 1 (S6K1), eukaryotic translation initiation factor 4E (eIF4E)-binding proteins 1 and 2 (4E-BP1/2), UNC-5 like autophagy activating kinase (ULK1), and transcription factor EB (TFEB) (Cornu et al., 2013; Lamming and Sabatini, 2013; Settembre et al., 2013b; Caron et al., 2015; Kim and Guan, 2015; Saxton and Sabatini, 2017). Growth factors such as insulin stimulate mTORC1 through the PI3K/AKT pathway. Activated AKT phosphorylates tuberous sclerosis complex 2 (TSC2) to inhibit TSC1, a GTPase-activating protein (GAP) for the small GTPase Ras homologue enriched in brain (RHEB), while the active RHEB strongly enhances mTOR activity (Inoki et al., 2002; Manning et al., 2002; Potter et al., 2002; Inoki et al., 2003a; Saucedo et al., 2003; Stocker et al., 2003; Tee et al., 2003; Zhang et al., 2003). Amino acids activate mTORC1 through the RAS-related GTP-binding protein (RAG) family of small GTPases, which promote the translocation of mTORC1 from the cytoplasm to the surface of lysosomes, where mTORC1 is activated by RHEB (Kim et al., 2008; Sancak et al., 2008; Sancak et al., 2010; Cornu et al., 2013; Saxton and Sabatini, 2017). Intracellular energy levels also regulate mTORC1 activity via TSC or RAPTOR in response to AMP-activated protein kinase (AMPK), or via the RAG GTPases in an AMPK-independent manner (Inoki et al., 2003b; Shaw et al., 2004; Gwinn et al., 2008; Kalender et al., 2010; Efeyan et al., 2013).

In contrast, mTORC2, which is insensitive to acute treatment with rapamycin but can be disrupted by chronic rapamycin treatment, responds to growth factors to modulate metabolism and cytoskeletal organization by activating AGC family kinases, including AKT, SGK1, and PKCα (Cornu et al., 2013; Saxton and Sabatini, 2017). mTORC2 phosphorylates AKT at Serine 473 in response to growth factor signaling and thereby enhances mTORC1 activity (Sarbassov et al., 2005; Cornu et al., 2013; Saxton and Sabatini, 2017). In addition, mTORC1 negatively regulates mTORC2 activity via phosphorylation of IRS1, Grb10, and mSIN1 (Harrington et al., 2004; Shah et al., 2004; Hsu et al., 2011; Yu et al., 2011; Cornu et al., 2013; Liu et al., 2013; Saxton and Sabatini, 2017). Although mTORC2 also regulates lipid homeostasis, much less is known about the substrates of mTORC2 than of mTORC1. Moreover, mTOR signaling regulates many metabolic and physiological processes, including protein synthesis, nucleotide synthesis, glucose metabolism, and lipid metabolism (Cornu et al., 2013; Caron et al., 2015; Saxton and Sabatini, 2017). In this review we focus on recent findings about the signaling mechanisms downstream of mTORC1 that regulate lipid metabolism in the liver.

## LIPID METABOLISM IN THE LIVER

As a major site of lipogenesis and lipid oxidation, the liver is a central organ in lipid metabolism, while impaired hepatic lipid metabolism is tightly correlated with obesity, diabetes, and fatty liver disease (Samuel and Shulman, 2012; Rui, 2014). In the fed state, when carbohydrates are abundant, the liver converts glucose into fatty acids (FAs) by *de novo* lipogenesis (Rui, 2014; Wang et al., 2015). Depending on the metabolic status, hepatocytes also obtain FAs from lysosomes by autophagy, and can also take up FAs from the bloodstream, which are released from adipose tissue and digested food in the gastrointestinal tract. FAs are then processed to triacylglycerols (TAGs) and cholesterol esters for storage during feeding or metabolized to produce energy during fasting (Rui, 2014; Wang et al., 2015).

Hepatic lipogenesis includes *de novo* lipogenesis of FAs from acetyl-CoA or malonyl-CoA and further processing to TAGs. Lipogenesis is catalyzed by the rate-limiting enzymes acetyl-CoA carboxylase (ACC) and fatty acid synthase (FAS), both of which are transcriptionally controlled by various transcriptional regulators in response to nutrients and hormones, including sterol response element-binding protein (SREBP) family members, carbohydrate-responsive element binding protein (ChREBP), and nuclear receptors (PPARγ, FXR, and LXR) (Rui, 2014; Wang et al., 2015). Insulin is the primary hormone that drives hepatic lipogenesis, while PI3K/AKT signaling is required for both inhibition of gluconeogenesis and stimulation of lipogenesis (Rui, 2014; Wang et al., 2015). However, it is paradoxical that both gluconeogenesis and lipogenesis are enhanced in obese and/or diabetic models, suggesting that lipogenesis is selectively resistant to insulin. The results from Brown and Goldstein’s lab show that inhibition of mTORC1 by rapamycin in primary rat hepatocytes and in rat liver tissues blocks insulin-stimulated lipogenesis, but has no effect on insulin-inhibited gluconeogenesis, indicating that mTORC1 is the point at which the insulin signaling pathway bifurcates to promote lipogenesis and inhibit gluconeogenesis (Li et al., 2010).

During fasting, glycogenolysis and gluconeogenesis are sequentially induced to produce glucose. Fasting also promotes lipolysis in adipose tissue, resulting in release of nonesterified fatty acids which are converted into ketone bodies in the liver through β-oxidation and ketogenesis (Rui, 2014; Wang et al., 2015). Autophagy is a conserved catabolic process that removes damaged macromolecules and organelles in response to stress and scarcity of nutrients (Rabinowitz and White, 2010; Mizushima and Komatsu, 2011). Recent studies have demonstrated that autophagy plays a critical role in lipid oxidation by shuttling lipid droplets to the lysosome for hydrolysis, a process named lipophagy (Singh et al., 2009; Rabinowitz and White, 2010; Mizushima and Komatsu, 2011).

## MTORC1 PROMOTES LIPOGENESIS

mTORC1 plays a critical role in promoting lipogenesis by regulating the expression of many lipogenic genes. One important family of transcription factors that controls lipid synthesis is the SREBPs. SREBPs belong to the family of basic helix-loop-helix-leucine zipper (bHLH-Zip) transcription factors. The SREBP family consists of three closely related members, SREBP1a, SREBP1c and SREBP2, among which SREBP1c and SREBP2 are the major isoforms expressed in the liver (Horton et al., 2002; Goldstein et al., 2006; Ferre and Foufelle, 2007). SREBP1 is a master transcriptional regulator of insulin-stimulated fatty acid synthesis, whereas SREBP2 mainly controls cholesterol synthesis (Horton et al., 2002; Goldstein et al., 2006; Ferre and Foufelle, 2007). SREBPs reside in the endoplasmic reticulum (ER) as inactive precursors with a complex including the sterol cleavage activating protein (SCAP) and insulin-induced gene (INSIG). Upon sensing insulin stimulation or sterol depletion, the SREBP/SCAP complex disassociates from INSIG and binds to Sec24, a subunit of the COPII complex, and then buds from the ER. The N-terminus of SREBP (nSREBP), which is released after cleavage by site-1 protease (S1P) and S2P in the Golgi, shuttles to the nucleus and induces the expression of genes involved in cholesterol and fatty acid synthesis (Horton et al., 2002; Goldstein et al., 2006; Ferre and Foufelle, 2007).

mTORC1 promotes the trafficking, processing, and transcription of SREBPs (Fig. 1B). In 2008, Portsmann and colleagues were the first to show that rapamycin impairs the nuclear accumulation of SREBPs and downregulates the expression of lipogenic genes (Porstmann et al., 2008). The results from Manning’s lab further demonstrated that mTORC1 is necessary for SREBP activity (Duvel et al., 2010; Yecies et al., 2011). mTORC1 promotes hepatic lipogenesis by activating SREBP in an S6K1-dependent and S6K1-independent manner (Peterson et al., 2011; Owen et al., 2012; Caron et al., 2015; Han et al., 2015). Although the mechanism of S6K1-dependent activation of SREBP is unclear, the S6K1-independent activation of SREBP involves inhibition and phosphorylation of CRTC2 (CREB regulated transcription coactivator 2) and Lipin-1 (Peterson et al., 2011; Han et al., 2015). CRTC2, a master regulator of gluconeogenesis, competes with Sec23A, a subunit of the COPII complex, to interact with Sec31A, another COPII subunit, thus disrupting SREBP1 transport from the ER to the Golgi. During feeding, mTOR is activated by insulin and/or amino acids, and then phosphorylates CRTC2, thereby attenuating the inhibitory effect of CRTC2 on COPII-dependent SREBP1 maturation (Han et al., 2015).

mTORC1 also regulates the SREBP transcriptional network at the transcriptional level via the negative regulation of Lipin-1, a phosphatidic acid phosphatase required for glycerolipid biosynthesis (Peterson et al., 2011). When phosphorylated by mTOR, Lipin-1 resides in the cytoplasm, while the dephosphorylated Lipin-1 shuttles to the nucleus. Nuclear Lipin-1 promotes the association of SREBPs with the nuclear matrix and inhibits their ability to bind SRE-containing lipogenic genes (Peterson et al., 2011). It should be noted that mTORC1 signaling is essential, but not sufficient, to activate SREBP-dependent lipogenesis in the liver. Since mTORC1 has positive regulatory roles in lipid synthesis, it was expected that liver-specific *Tsc1* null mice would develop severe hepatosteatosis. However, *Tsc1* null mice were protected against age- and diet-induced hepatic lipid accumulation (Yecies et al., 2011). It is possible that the constitutively active mTORC1 negatively feeds back to AKT, thereby enhancing the expression of *Insig2a*, a negative regulator of SREBPs in the liver, and finally inhibiting the processing of SREBPs (Yecies et al., 2011).

As discussed above, mTORC1 enhances lipogenesis via the positive regulation of SREBPs. It remains unclear whether mTORC1 affects other transcriptional regulators of lipogenic genes. Since mTORC1 is over-activated due to the enhanced levels of branched chain amino acids in obese models (Um et al., 2004; Khamzina et al., 2005; Han et al., 2015), these recent insights into the regulation of lipogenesis by mTORC1 provide us with a better picture to understand the selective insulin resistance that underlies the enhanced lipogenesis and gluconeogenesis in obese animals.

## MTORC1 INHIBITS LIPOPHAGY

mTORC1 inhibits lipophagy mainly through the inhibition of autophagy and lysosome biogenesis (Fig. 1C). mTORC1 inhibits the autophagy-initiating UNC-51-like autophagy activating kinase (ULK) complex by phosphorylating complex components including autophagy-related gene 13 (ATG13) and ULK1/2. mTORC1 phosphorylates ULK1 and prevents the phosphorylation of ULK1 by AMPK. Moreover, mTORC1 phosphorylates ATG14L, a component of the VPS34 complex, thereby inhibiting the kinase activity of VPS34 and blocking autophagosome formation (Ganley et al., 2009; Hosokawa et al., 2009; Jung et al., 2009; Kim et al., 2011; Yuan et al., 2013; Shimobayashi and Hall, 2014; Kim and Guan, 2015).

The lysosome is a central organelle for energy metabolism and for nutrient sensing and recycling in response to starvation or nutritional stress (Xu and Ren, 2015). mTORC1 also inhibits lysosome biogenesis at the transcriptional level by the direct phosphorylation of TFEB. TFEB, a member of the MiTF/TFE (microphthalmia-associated transcription factor) family of transcription factors that includes MITF, TFE3 (transcription factor binding to IGHM enhancer 3), TFEB and TFEC, controls the expression of an array of genes involved in lysosome biogenesis and autophagy (Settembre et al., 2013b; Raben and Puertollano, 2016).

Under fed conditions, TFEB is phosphorylated by mTOR at multiple serine residues and sequestered in the cytoplasm by binding to 14-3-3 (Pena-Llopis et al., 2011; Martina et al., 2012; Roczniak-Ferguson et al., 2012; Settembre et al., 2012). In contrast, starvation induces calcium release from lysosomes through MCOLN1 (Mucolipin 1), further activating the calcium-dependent protein phosphatase 2B (PP2B/calcineurin), and thereby promoting TFEB dephosphorylation (Medina et al., 2015). In addition, FGF21, a fasting-induced hormone, promotes lipid oxidation and ketogenesis, and enhances PP2A-dependent dephosphorylation of TFEB (Chen et al., 2017). Dephosphorylated TFEB shuttles to the nucleus and directly binds to a 10-base pair motif known as a CLEAR (coordinated lysosomal expression and regulation) element, which is enriched in the promoter of numerous autophagic and lysosomal genes (Sardiello et al., 2009; Settembre et al., 2011; Settembre et al., 2013b). By activating the transcription of these genes, TFEB promotes autophagy and lysosome biogenesis. Furthermore, TFEB has been shown to promote lipid oxidation by upregulating the expression of PPARα and PPARγ coactivator 1 α (PGC1α) (Settembre et al., 2013a), which are master regulators of lipid oxidation and mitochondrial biogenesis (Handschin and Spiegelman, 2006; Fan and Evans, 2015). HLH-30, an orthologue of TFEB in *C*. *elegans* has a similar effect on lipid metabolism (O’Rourke and Ruvkun, 2013), indicating that the roles of TFEB in lipid metabolism are conserved. Therefore, TFEB orchestrates lipophagy by coordinating lysosome biogenesis, autophagy, lipid oxidation, and mitochondrial function.

TFE3, another member of the MiTF/TFE family, is also phosphorylated by mTORC1 and regulates autophagy and lysosome biogenesis in starved cells by binding to the CLEAR elements of autophagic and lysosomal genes (Martina et al., 2014; Raben and Puertollano, 2016). Interestingly, ZNF306 (ZKSCAN3), a transcriptional repressor of zinc finger transcription factors, is phosphorylated by mTORC1 and stays in the nucleus to inhibit the expression of autophagic and lysosomal genes in fed cells (Chauhan et al., 2013).

Interestingly, mTORC1 is inactivated during autophagy initiation and is then reactivated, probably by the increased amino acid levels that are generated by lysosomal degradation after long periods of starvation. The reactivated mTORC1 is important for lysosomal reformation (Yu et al., 2010). Also, lysosomal positioning is critical for the kinase activity of mTORC1 (Korolchuk et al., 2011). The cooperation of mTORC1 with the regulators of the autophagic-lysosomal pathway ensures an efficient autophagy flux in response to different environmental cues. Defective functioning of the autophagic-lysosomal pathway, dysregulated mTORC1 signaling, and impaired lipid metabolism in the liver affect each other and may further exacerbate vulnerable lipid homeostasis and insulin sensitivity in obesity (Um et al., 2004; Khamzina et al., 2005; Yang et al., 2010; Samuel and Shulman, 2012; Han et al., 2015; Martinez-Lopez and Singh, 2015).

## CONCLUSIONS

The identification of novel regulators has further strengthened our knowledge of the basic layout of mTORC1 signaling and its central role in lipid metabolism by promoting lipogenesis and inhibiting lipophagy in the liver. Even though rapamycin has been shown to increase lifespan and to protect against cancer, side effects such as dyslipidemia may limit its clinical usefulness. Therefore, identifying *bona fide* mTOR substrates and their molecular roles in lipid metabolism is a promising approach to generate new compounds to target these mechanisms in the future.
